# Musical training, individual differences and the cocktail party problem

**DOI:** 10.1038/srep11628

**Published:** 2015-06-26

**Authors:** Jayaganesh Swaminathan, Christine R. Mason, Timothy M. Streeter, Virginia Best, Gerald Kidd, Jr, Aniruddh D. Patel

**Affiliations:** 1 Department of Speech, Language and Hearing Sciences, Boston University, Boston, MA; 2 Department of Psychology, Tufts University, Medford, MA.

## Abstract

Are musicians better able to understand speech in noise than non-musicians? Recent findings have produced contradictory results. Here we addressed this question by asking musicians and non-musicians to understand target sentences masked by other sentences presented from different spatial locations, the classical ‘cocktail party problem’ in speech science. We found that musicians obtained a substantial benefit in this situation, with thresholds ~6 dB better than non-musicians. Large individual differences in performance were noted particularly for the non-musically trained group. Furthermore, in different conditions we manipulated the spatial location and intelligibility of the masking sentences, thus changing the amount of ‘informational masking’ (IM) while keeping the amount of ‘energetic masking’ (EM) relatively constant. When the maskers were unintelligible and spatially separated from the target (low in IM), musicians and non-musicians performed comparably. These results suggest that the characteristics of speech maskers and the amount of IM can influence the magnitude of the differences found between musicians and non-musicians in multiple-talker “cocktail party” environments. Furthermore, considering the task in terms of the EM-IM distinction provides a conceptual framework for future behavioral and neuroscientific studies which explore the underlying sensory and cognitive mechanisms contributing to enhanced “speech-in-noise” perception by musicians.

Intensive musical training places significant demands on auditory processing (e.g., in making subtle distinctions between sounds in terms of pitch, timing, and timbre) and on cognitive abilities such as auditory attention and working memory. These demands are not unique to music. Speech perception, for example, also depends on detailed auditory analysis operating in concert with working memory and auditory attention^1^. The shared demands of musical training and speech perception may rely on partly overlapping brain mechanisms: growing evidence suggests that the brain networks involved in music and speech processing are not entirely segregated within the cerebral cortex, and may in fact have a significant degree of overlap^2^,^3^,^4^,^5^. This raises a fundamental question: are linguistic and musical abilities related in significant ways, or do they constitute largely distinct mental faculties, as suggested by some theorists^6^?

One way to address the question is to compare musically trained and untrained individuals on language processing tasks. If musically-trained individuals show benefits on these tasks, this would suggest neurobiological connections between music and speech processing. This could arise because 1) individuals who are innately advantaged in certain auditory and cognitive processes shared by music and speech are attracted to musical training, 2) musical training enhances speech processing via experience-dependent neural plasticity in brain networks shared by speech and music, or because of a combination of 1) and 2)^7^,^8^.

There are now numerous studies comparing musically trained and untrained individuals across a variety of language tasks. For some abilities, including speech intonation perception^9^,^10^, vocal affect discrimination in sentences^11^,^12^, and production and perception of second language phonological contrasts^13^,^14^, multiple studies have found musical training to be associated with enhanced speech processing. One area where research has produced less consistent results, however, concerns speech perception in “noise” (meaning, generally, unwanted sounds not limited to white noise or speech-shaped noise). This is an important ability in everyday life since speech is often heard in the context of other sounds, and is also an ability in which normal-hearing individuals can vary widely^15^.

The idea that musicians might show benefits in speech-in-noise perception seems plausible. In the practice of their art, musicians depend upon their ability to listen selectively to individual instruments within a musical ensemble and to shift the focus of attention from one instrument to another at will. This bears a striking similarity to the problem of attending to a specific human voice among several competing voices, an extensively-studied problem known as the “cocktail party” problem^16^,^17^. Conversing in a “cocktail party” type of environment has been shown to be extremely challenging for listeners with sensorineural hearing loss^18^, for cochlear implantees^19^, and even for some listeners with clinically normal hearing^15^.

There are, of course, several differences between selective listening in musical and linguistic contexts. For example, members of a musical ensemble are typically playing the same piece (although different instruments may be playing different parts), while the cocktail party problem involves selecting a given talker from various independent conversations. However, to the extent that both situations place demands on the capacity for selective listening in a complex auditory scene, and these demands engage brain networks shared by music and speech processing, then one might expect musicians to have an enhanced ability to select and attend to a target talker in the presence of competing (masking) talkers.

To date, research on speech perception in multiple-source environments by musicians has produced equivocal results. On the one hand several studies have reported small but statistically significant benefits for musicians on standard tests of speech-in-noise perception. For example, Parbery-Clark *et al.*^20^, demonstrated a small but significant performance advantage for young adult musicians over non-musicians in two clinical tests of speech understanding in noise (overall effect size <1 dB between groups). More recently, however, two other studies (Ruggles *et al.*^21^; Boebinger *et al.*^22^) found no benefit for musicians in tests of speech-in-noise perception. Given these inconsistent results, further research on this topic is warranted because of its potential theoretical and practical significance. In terms of basic research, if musicians show clear advantages for hearing speech in noise, this would offer researchers a useful population for exploring the mechanisms (sensory and cognitive) that contribute to better speech-in-noise perception. This in turn could help hearing scientists understand the factors underlying the large individual differences mentioned above. From a practical perspective, if musical training actually causes improvements in speech-in-noise perception, this would have significant implications for designing training programs to enhance this ability in normal and clinical populations^7^.

The current study examines speech perception in musically trained and untrained individuals, using a multiple-talker masking approach. We focus not on questions of causality, which require longitudinal studies with random assignment to musical vs. nonmusical training, but rather on attempting to determine whether musicians show benefits for selective listening in a cocktail-party like listening task. Unlike most previous studies, we use competing sounds that consist of intelligible sentences that are spatially separated from the target sentence. This emulates an ecologically realistic situation in which one seeks to understand an interlocutor whom one is facing directly while trying to ignore nearby speakers. To help distinguish between the different factors which contribute to masking in such situations, we separately manipulate two types of masking caused by the interfering speech: informational and energetic masking (henceforth, IM and EM). EM occurs when maskers overlap in time and frequency with the target, producing competition for representation at the auditory periphery (e.g., “sensory interference”). IM occurs when maskers are highly similar to and/or confusable with the target, thus producing competition at physiological sites beyond the auditory periphery (e.g, “cognitive interference”). Using Gaussian white noise or speech-shaped noise to mask speech, for example, creates high EM but little IM, since there is no other intelligible signal competing for cognitive processing. By using speech as the masking stimulus we create both EM and IM, but crucially, we can manipulate the maskers in specific ways to vary the amount of IM in different conditions, from very high to very low.

The modulation of IM in our stimuli is based on manipulating both the spatial location and the intelligibility of the masking speech. In terms of spatial location, it has been demonstrated that the intelligibility of target speech is improved considerably when the competing maskers are spatially separated from the target^23^,^24^, or appear to be separated from the target^25^,^26^, relative to the case when all of the sounds arise from the same location, an effect referred to as “spatial release from masking” (SRM). It also has been shown that the IM component of speech-on-speech masking may play a critical role in determining the magnitude of SRM (e.g., reviewed in^27^). In other words, much of the benefit listeners receive from spatially separating the target speech from the masker speech seems to be due to cognitive factors producing a release from IM (e.g., the ability of the listener to focus on a target signal and suppress the cognitive/linguistic processing of distractor signals) rather than exclusively to sensory factors producing a release from EM (e.g., reduction of within-channel competition for representation of the target^28^).

In terms of intelligibility, we manipulated the IM produced by the masking speech by either playing it forward (in which case it was normal and fully intelligible) or by reversing its time-domain signal (rendering it unintelligible). The comparison of performance with these two maskers has the advantage that they have very similar spectrotemporal structures (see Fig. 1) and thus are expected to produce equivalent amounts of EM while differing substantially in the amount of IM they produce. However, it should be noted that the relative benefit for target speech intelligibility produced by time-reversing masker speech may depend crucially on the specific procedures used (e.g., the speech corpus, the way the target speech is designated separate from the masker speech, other segregation cues present such as talker sex differences, etc; see recent review in^29^) and some studies have reported little or no effect of masker time reversal^30^,^31^,^32^. Generally, if the procedures involved produced little IM for forward masker speech, then time-reversal would likely not provide much of a benefit. Here, we used neural modeling of auditory peripheral processing to verify that, for the stimuli used in this study, forward and reversed maskers produced similar EM of the target temporal features (see supporting information for details) and therefore any differences in the masking they produced could reasonably be attributed to differences in IM.

There are four conditions in our study. In all conditions, the target is a short intelligible sentence (e.g., “Jane saw two red shoes”) coming from directly ahead of the participant. The target is always presented with two other similar sentences (spoken by different speakers) which serve as maskers. In conditions 1 and 2 the maskers are intelligible and are either colocated with the target (condition 1) or spatially separated from it (condition 2). In conditions 3 and 4 the maskers are unintelligible (time-reversed) and are again either colocated with the target (condition 3) or spatially separated from it (condition 4). This results in set of conditions in which EM is very similar but in which IM is gradually reduced from very high in condition 1, intermediate in conditions 2 and 3, and very low in condition 4.

We predicted that if musicians showed a benefit for hearing speech in noise, the degree of this benefit would be strongly modulated by the amount of IM created by the maskers. This prediction was based on prior research showing musician advantages on auditory cognitive tasks using nonlinguistic stimuli^33^,^34^. It was also based on research with nonlinguistic stimuli (e.g., tone bursts) showing that musicians are better at concurrent sound segregation and less susceptible to IM than non-musicians^35^,^36^. The current work built on this prior work, but employed intelligible, spatialized speech as the key stimulus, in order to determine if musicians showed advantages for speech-in-noise perception in more ecologically valid situations.

## Results

In each trial of the psychophysical task three simultaneous streams of speech were presented. The target sentence was always presented from directly ahead (0° azimuth), while two concurrent masker sentences (presented as forward or reversed speech) were presented either from directly ahead (colocated configuration) or from different directions (separated configuration): one 15° to the left and one 15° to the right as simulated by head-related transfer functions (HRTFs). The listener was instructed to identify the words comprising the target while ignoring the maskers (see methods for details). The masker levels were fixed at 55 dB SPL and the target level was adjusted adaptively based upon the listener response to achieve a 50% correct identification threshold. Neural modeling of auditory peripheral processing across conditions of this study (forward vs reversed maskers) confirmed that the amount of energetic masking or “sensory interference” between maskers and target was similar and closely matched (see supporting information).

Figure 2 shows target-to-masker ratios (TMRs) at threshold (calculated as the target level minus the masker level in dB) for individual subjects (panel A) and group means (panel B) for musicians and non-musicians. The TMRs are plotted for colocated and separated configurations for both forward and reversed maskers. Lower TMRs correspond to less masking.

For the forward thresholds, a two-way mixed-design ANOVA revealed a significant effect of spatial configuration [*F*(1,22) = 396.9, *p* < 0.001, η^2^ = 0.947], listener group [*F*(1,22) = 13.9, *p* = 0.001, η^2^ = 0.388], and a significant interaction [*F*(1,22) = 13.4, *p* = 0.001, η^2^ = 0.379]. When the forward speech maskers were colocated with the target, mean thresholds were similar for musicians (M) and non-musicians (NM) (M: 2.6 dB, NM: 3.7 dB). However, the musicians achieved substantially lower thresholds than non-musicians when the forward maskers were spatially separated from the target (M: −15.1 dB, NM: −8.5 dB). Independent samples two-tailed *t*-tests confirmed that the musicians and non-musicians had similar colocated thresholds [*t*(22) = −1.1, *p* = 0.3], but had significantly different separated thresholds [*t*(22) = −4.5, *p* < 0.001].

Among the musicians, there was no significant relationship between the separated thresholds with forward speech maskers and duration of musical training or age of onset of musical training. Among non-musicians, large individual differences were observed in the separated thresholds, ranging over 15 dB (from 0 to −15 dB), compared to a range of just 5 dB in musicians (−13 to − 18 dB) (Fig. 2A). The simple subtraction of the thresholds in the two configurations indicates that musicians achieved a substantially larger SRM than non-musicians (M: 17.7 dB, NM: 12.2 dB; see Fig. 2C). Independent samples two-tailed *t*-tests confirmed that the difference in SRM between musicians and non-musicians was significant [*t*(22) = 3.7, *p* = 0.001].

For the reversed thresholds, a two-way mixed design ANOVA revealed a significant effect of spatial configuration [*F*(1,22) = 80.5, *p* < 0.001, η^2^ = 0.785], listener group [*F*(1,22) = 7.6, *p* = 0.011, η^2^ = 0.258], and a significant interaction [*F*(1,22) = 5.4, *p* = 0.03, η^2^ = 0.197]. When the reversed speech maskers were colocated with the target, the musicians achieved significantly lower thresholds than non-musicians (M: −16.2 dB, NM: −12.8 dB; *t*(22) = −2.9, *p* = 0.007). However, no difference between the groups was observed when the reversed maskers were spatially separated (M: −18.9 dB, NM: −17.3 dB; *t*(22) = −1.9, *p* = 0.062). Interestingly, mean SRM was significantly higher for the non-musicians than musicians (M: 2.7 dB, NM: 4.6 dB; *t*(22) = −2.3, *p* = 0.03; Fig. 2C) which was largely due to the higher (poorer) TMRs in the colocated condition in non-musicians relative to the similar TMRs in the separated condition for the groups. Individual differences were still observed in the colocated and separated thresholds across the two groups ranging from −8 dB to −21 dB for the colocated configuration and −13 dB to −22 dB for the separated configuration. However, the overall range of individual differences in separated thresholds was lower with reversed maskers compared to the differences observed with forward maskers (~9 dB vs ~18 dB).

To examine patterns of individual performance across listeners, we examined correlations between separated thresholds for forward versus reversed maskers (Fig. 3). Results reveal that the thresholds across the two masker types are correlated [*r*(22) = 0.59, *p* = 0.002] with the better listeners (musicians and some non-musicians) achieving lower threshold values for both masker types. However, the slope of the least-squares fit was shallow (0.26) as the poorest listeners in the non-musicians group (with higher separated thresholds in the forward condition) were still able to achieve better (lower) thresholds when the maskers were reversed. This demonstrates that the poor performers, who did not benefit as much from spatializing the maskers when presented as forward speech, were able to make use of spatial cues when IM was reduced with the reversed maskers.

## Discussion

In this study, we found that musicians performed significantly better than non-musicians on a task that emulated the classical “cocktail party problem”, in which a listener attempts to understand one talker while ignoring intelligible speech from other talkers who are spatially separated from the target talker. In contrast to prior work, in which either a small or no benefit was observed for speech-in-noise perception in musicians (e.g., <1 dB in Parbery-Clark *et al.*^20^; no benefit in, Ruggles *et al.*^21^, and Boebinger *et al.*^22^), here a substantial benefit (~6 dB; see Fig. 4) was obtained for musicians when the masking sounds were intelligible speech that was spatialized relative to the target. Parbery-Clark *et al.*^20^, tested musicians and non-musicians in two clinical measures of speech perception in noise with the speech target masked by either a speech shaped noise (HINT) or multi-talker babble (Quick SIN). The speech shaped noise masker was presented either colocated with the target (0°) or spatialized relative to the target location (at 90° to the right or left). Parbery-Clark *et al.*^20^, reported a small (overall effect size <1 dB) yet significant benefit of musical training in both the clinical speech tests when the maskers were colocated with the target but reported no benefit when the maskers were separated in space. It should be noted that both of the maskers used by Parbery-Clark *et al.*^20^, were mostly energetic in nature. Hence, it is not entirely surprising that musicians and non-musicians achieved comparable scores when the (speech shaped noise) maskers were spatialized, given the small difference between the groups (<1 dB) in the more difficult baseline condition in which the maskers were colocated with the target. Ruggles *et al.*^21^, tested musicians and non-musicians with voiced and whispered speech, in either continuous or fluctuating noise, in order to investigate whether the musicians’ improved performance as reported by Parbery-Clark *et al.*^20^, was due to more efficient coding of periodicity in normal speech. However, they found no significant effect of musicianship with colocated maskers even for conditions similar to those used by Parbery-Clark *et al.*^20^. Boebinger *et al.*^22^ tested musicians and non-musicians using four different kinds of maskers that varied in the amount of energetic and informational masking in order to gain a better mechanistic understanding of the musicianship advantage: 1) natural speech, 2) spectrally rotated speech, 3) speech-shaped steady noise and 3) speech-shaped amplitude modulated noise. Boebinger *et al.*^22^ found no advantage for musicians’ speech perception in noise compared to non-musicians and this did not vary by masker type. However, in their study, for the condition designed to produce high informational masking (natural speech maskers) the sex of the target speech (female) was different than that of the masker (male). This meant that the informational masking effects were reduced compared to conditions with same sex speakers^37^ which would make the task easier for both musicians and non-musicians. In our study, by manipulating the location and intelligibility of the masking speech (using same sex speakers), we were able to systematically vary informational masking while keeping energetic masking constant. Overall, the results from this and previous studies suggest that the characteristics of speech maskers and the amount of informational masking can influence the differences found between musicians and non-musicians in multiple-talker “cocktail party” like environments.

### Musical training and spatial hearing

Although musicians demonstrated substantially better speech-in-noise perception than non-musicians when the target speech was masked by two intelligible, spatially separated speech maskers, they showed no difference from non-musicians when the same maskers were colocated with the target. Thus the overall difference in spatial release from masking (SRM) in the two groups (~5 dB, cf. Fig. 2C, forward maskers) was driven almost entirely by a musician benefit in the spatially-separated condition. The colocated configuration is high in both energetic and informational masking, and it appears that this difficult baseline condition requires the target to be the loudest source in the mixture in order for it to be understood (i.e., target-to-masker ratios >0 dB). However, spatially separating the maskers takes the listeners out of this TMR region by reducing IM, which in turn may have helped in segregating the target stream from masker streams^24^,^38^,^39^. This enabled both musicians and non-musicians to achieve much lower thresholds, in which the target was intelligible even when quieter than the maskers (i.e., average target-to-masker ratios <0 dB). It is in this condition that musicians achieve substantially lower thresholds than non-musicians (difference of ~6 dB). This may be attributed to their enhanced ability to suppress irrelevant background sounds, which suggests that musicians are less affected by informational masking than non-musicians. This conclusion is consistent with prior work using basic nonlinguistic auditory stimuli, which demonstrated that musicians are less susceptible to IM^35^ and show superior performance on auditory attention tasks^33^, perhaps reflecting superior “analytic” listening abilities compared to non-musicians^35^. Our results suggest that the benefits demonstrated with basic auditory stimuli generalize to speech perception in the presence of interfering speech maskers that produce informational masking.

When the maskers were presented as reversed speech colocated with the target, the musicians achieved significantly lower thresholds than non-musicians. In the colocated configuration, in which the amount of EM was high and comparable to intelligible (forward) maskers (see supplementary information), the amount of IM was lower than in the intelligible speech conditions because the distractors were no longer meaningful. In this condition, musicians achieved significantly lower thresholds than non-musicians (difference ~3 dB), which may be related to their enhanced sound source/stream segregation abilities^36^. However, when the reversed maskers were spatially separated from the target, all of the listeners achieved substantially lower thresholds, presumably due to further release from IM (compared to the colocated configuration) and ease of the task. This result is consistent with previous studies that have shown a small but significant performance advantage (<1 dB) in young adult musicians (vs non-musicians) in understanding speech in the presence of speech shaped noise maskers (purely energetic maskers low in IM) when colocated with the target^20^ (also see^21^) but no difference in performance when the maskers were spatially separated^20^.

### Influence of informational masking in explaining individual differences

Consistent with previous studies^15^, large individual differences were observed in how effectively normal-hearing listeners were able to identify a speech target in the presence of spatially separated speech maskers. In fact, the individual differences were quite dramatic across musicians and non-musicians for separated thresholds with two intelligible maskers (range ~20 dB). It has been suggested that these individual differences can be related to peripheral sensory coding deficits. However, and crucially, the poorer performers in our tasks with the intelligible maskers improved considerably when the spatialized maskers were reversed and less confusable with the target (range of separated thresholds across the groups ~9 dB). In this condition, the musicians and non-musicians both achieved substantially lower thresholds and performed comparably (Fig. 4). Importantly, in the two conditions with spatialized maskers, spatial cues and energetic masking remained the same and the primary difference was the intelligibility of the maskers. This suggests that the listeners in the non-musician group with poorer separated thresholds with forward speech maskers did not have a complete inability to spatialize and segregate the maskers from the target *per se*. This seems inconsistent with an explanation based purely on sensory coding deficits/differences across these individuals. Rather, the individual differences may be explained (at least partly) by differences in susceptibility to IM. This conclusion is consistent with other studies that have used basic auditory stimuli to demonstrate that informational masking is often accompanied by large differences in performance between normal-hearing listeners^40^,^41^. That said, it would be useful for future work to compare spatial acuity directly in musicians and non-musicians, in order to determine how any differences in this ability might contribute to the benefit found in musicians in speech-in-noise perception in multiple-talker “cocktail party” like situations. While our study focused on young adults, it will also be interesting to determine whether the results seen here generalize to musician vs. non-musician populations among older adults, who may have more sensory coding deficits than younger adults^42^. Interestingly, recent research has found that musicians show less age-related decline in speech-in-noise tasks than non-musicians^43^, though spatial hearing was not tested.

### Conclusions and future directions

Overall, our results suggest that the differences in spatial hearing ability between musicians and non-musicians are determined in part by the characteristics and amount of IM present in the competing speech maskers. It is important to note that further empirical work is needed to determine the extent to which our results generalize to other listening situations. For example, Carey *et al.*^44^ found that musicians and non-musicians performed similarly in an “environmental auditory scene analysis task” which required nonlinguistic sounds (such as animal sounds) to be detected in natural-sounding auditory scenes (such as farmyards). Thus there is a strong need to test variations of the current paradigm, e.g., varying the speech corpora/materials, number and gender of speakers, degree of spatial location, and manner of manipulating IM. In particular, we suggest that future work use different approaches to vary IM, with an eye toward ecological validity. With respect to understanding the neural mechanisms, an important direction for future work is to manipulate IM and study behavioral and neural data on speech perception in noise, using neural measures of subcortical and cortical processing to try to disentangle sensory and cognitive processing^45^,^46^,^47^,^48^,^49^.

As with any cross-sectional study, we cannot infer from the current results that musical training caused improvements in the ability to hear speech in noise^50^. The issue of causality can only be addressed by longitudinal training studies with random assignment of individuals to musical training vs. to other forms of training (or no training), guided by specific hypotheses for how and why musical training would influence speech processing^7^. Thus it is important to note that the focus of the current work is not on issues of causality, but on investigating associations between musical training and enhanced speech perception in a multi-talker environment, as well as the bases of these enhancements.

More generally, we suggest that our approach of testing normal-hearing listeners with varying listening abilities (i.e., musicians vs. non-musicians) in ecologically-realistic conditions (spatial hearing) using speech maskers with varying amounts of IM (such as forward vs reversed speech) can further our understanding of the relative roles of cognitive and sensory factors in explaining individual differences in hearing speech in noise.

## Materials and Methods

### Subjects

Twelve musicians (mean age = 23.0 years; SD = 2.8 years) and 12 non-musicians (mean age = 20.3 years; SD = 1.1 years) with normal hearing (defined as <=20 dB HL pure-tone thresholds from 250 to 8,000 Hz) and no history of neurological disorders participated in the study. All subjects were native speakers of American English. Subjects completed a musical history form that assessed beginning age and length of musical training (at the time of the study), practice frequency and intensity, as well as how often they listened to music. Subjects who were categorized as musicians had at least 10 years of formal musical training (10–18 years), and most musicians currently practiced at least 5 hours a week (see Table 1 for further details). Nearly all individuals categorized as musicians were enrolled in the School of Music at Boston University. Subjects who were categorized as non-musicians had minimal to no formal musical training and did not report currently playing a musical instrument or routinely participating in any musical activity (other than passive listening). No tests of general cognitive measures were conducted between the groups. All experiments were performed in accordance with relevant guidelines and regulations with an approved Institutional Review Board protocol from the Boston University Human Research Protection Program. All subjects were fully informed about the goals of the study and provided written consent before their participation.

### Procedure

**Figure 1:**
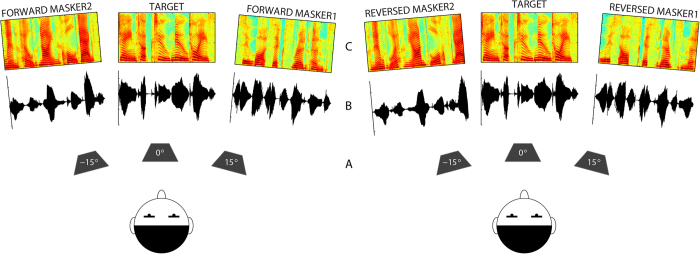
****A**: Speaker locations relative to listener; **B**&**C**: Example target and masker waveforms and spectrograms for forward and reversed speech. Target: “Jane took two new toys”; Forward masker1: “Sue bought six red pens”; Forward masker2: “Lynn held nine cold bags”.

**Figure 2:**
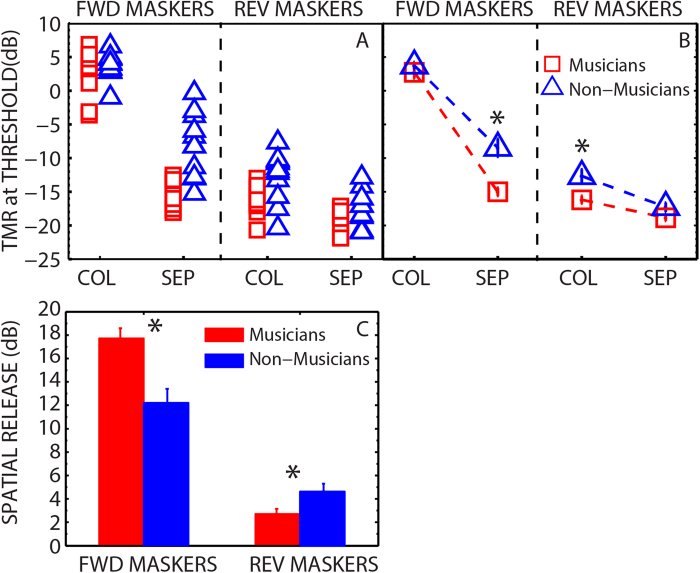
Musicians achieved substantially lower thresholds than non-musicians for hearing speech masked by interfering speech. Panel **A**: Individual target-to-masker ratio at threshold (TMR) for musicians (red squares) and non-musicians (blue triangles) measured in colocated and separated configurations. The left side of the panel shows results with forward (FWD) maskers, while the right side shows results with reversed (REV) maskers. TMR was calculated as the level of the target at adaptive threshold minus the fixed masker level (55 dB SPL). Panel **B**: Group mean TMRs for conditions shown in panel A. Panel **C**: Mean spatial release from masking (SRM = colocated – separated thresholds) for forward and reversed masker configurations measured from musicians and non-musicians. Error bars are ±1 standard error of the mean. *- Statistically significant group difference.

**Figure 3:**
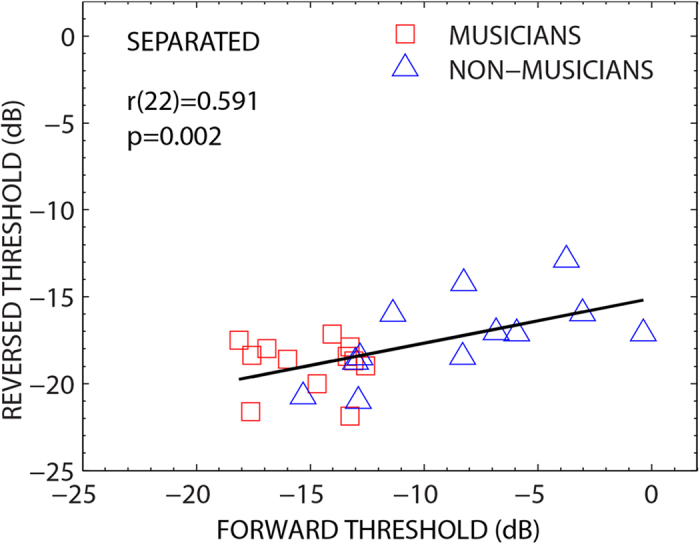
For separated target and maskers, thresholds were correlated across the two masker types (forward and reverse). However, listeners achieved lower thresholds with reversed maskers (low IM) than forward maskers (high IM). Scatter plot shows thresholds for forward and reversed maskers in the 2 masker separated configurations. Solid line shows least-squares fit to the data points.

**Figure 4:**
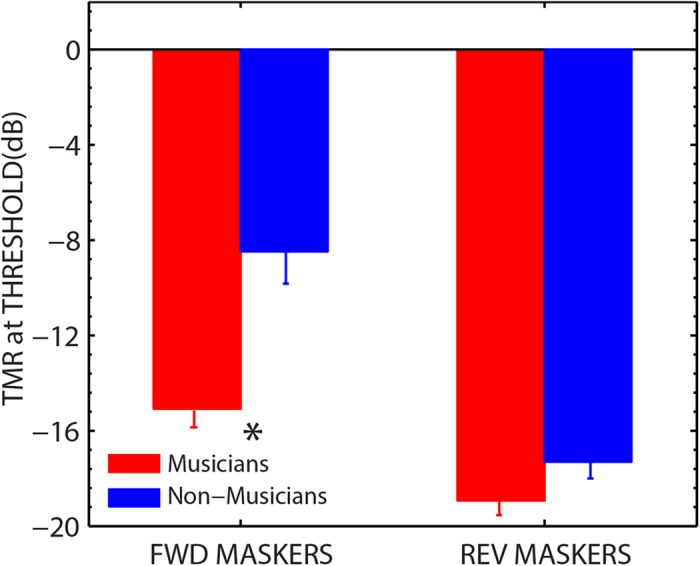
Musicians achieved substantially lower thresholds than non-musicians with forward separated maskers and not with reversed separated maskers. Plot shows group mean TMRs for musicians and non-musicians with spatialized maskers presented as forward (FWD) or reversed (REV) speech. Error bars are ±1 standard error of the mean. *- Statistically significant group difference.

On each trial, the target and masker were comprised of five-word sentences that were syntactically correct but not necessarily semantically meaningful. The sentences had the structure <name> <verb><number> <adjective> <object> and there were 8 possible words in each category^38^. One sentence was designated as the target and always contained the <name> call-sign “Jane” with other keywords being randomly selected from the available choices (e.g., Jane took two new toys). The masker sentences contained randomly selected <name> call-signs (excluding “Jane”) and keywords that differed from the target and from each other. The target and masker sentences were spoken by different female talkers selected at random on each trial from the seven available talkers. The masker sentences were either played naturally (forward condition) or were time-reversed on a word-to-word basis to render them unintelligible (reversed condition, see Fig. 1).

Stimuli were delivered via Sennheiser HD 280 PRO headphones to listeners seated in a double-walled sound-attenuating chamber (Industrial Acoustics Company). Digital stimuli were generated on a PC outside the booth and then fed through separate channels of Tucker-Davis Technologies System II hardware. Target and maskers were spatialized using KEMAR head-related transfer functions. The HRTF’s were obtained using tone sweeps recorded in a single-walled Industrial Acoustics Company sound booth (12 ft. × 14 ft. × 7.5 ft). Target speech was presented from 0° azimuth, and the maskers were presented either from the same location (colocated) or symmetrically separated in azimuth at ±15°.

On a given run, the maskers were fixed equal in level at 55 dB SPL and the level of the target was varied adaptively using a one-down one-up procedure that tracked the 50% correct point on the psychometric function (giving a threshold target-to-masker ratio, TMR). The target level was varied adaptively in 6 dB steps initially and then in 3 dB steps following the third reversal. Each run consisted of at least 25 trials and at least 9 reversals. Subjects were instructed to identify the keywords coming from the front uttered by the target talker. The possible responses were displayed orthographically on a computer screen. Subjects reported the perceived target keywords using the computer mouse to select the buttons showing the keywords on the screen. Correct answer feedback was provided during testing. Responses were counted as correct only if the listener successfully identified all four keywords correctly. Each listener was tested in 2 speech conditions (forward and reversed maskers) × 2 spatial configurations (colocated and separated) × 6 runs for each speech condition and spatial configuration totaling 24 runs which were completed in two sessions. The ordering of the runs was completely randomized across subjects. The first 2 runs for each condition was used as practice runs and were not included in the data analysis.

## Additional Information

**How to cite this article**: Swaminathan, J. *et al.* Musical training, individual differences and the cocktail party problem. *Sci. Rep.* **5**, 11628; doi: 10.1038/srep11628 (2015).

## Supplementary Material

Supplementary Information

## Figures and Tables

**Table 1:** List of musicians, age at onset of musical training, duration of musical training and primary instrument.

Subject	Age musical training began (yr)	Duration of musical training (yr)	Primary instrument
M1	6	15	Piano
M2	8	14	Voice
M3	7	15	Tuba
M4	2	19	Piano
M5	12	13	Flute
M6	10	12	Flute
M7	11	10	Bassoon
M8	14	10	Bass trombone
M9	5	17	Double bass
M10	10	18	Flute
M11	10	11	Clarinet
M12	7	11	Piano
