# Erratum: Musical training, individual differences and the cocktail party problem

**DOI:** 10.1038/srep14401

**Published:** 2015-09-25

**Authors:** Jayaganesh Swaminathan, Christine R. Mason, Timothy M. Streeter, Virginia Best, Gerald Kidd, Jr, Aniruddh D. Patel

Scientific Reports 5: Article number: 11628;10.1038/srep11628 published online: 06262015; updated: 09252015

In the Supplementary Information file originally published with this Article, there is a typographical error in Affiliation 2 “Department of Psychology, Tufts University, Medford, MA”, which was incorrectly given as “Department of Psychology, Tufts University, Malden, MA”. This error has been corrected in the Supplementary Information that now accompanies the Article.

