# Supplementary material for: Musical training, individual differences and the cocktail party problem
Source: Sci Rep. 2015 Jun 26;5:11628. doi: 10.1038/srep11628 (PMC4481518; doi:10.1038/srep11628)
Supplement: Supplementary Information [file srep11628-s1.pdf]

Supplementary Information

Musical training, individual differences and the cocktail party problem

Jayaganesh Swaminathan [a,1]

Christine R. Mason [a]

Timothy M. Streeter [a]

Virginia Best [a]

Gerald Kidd, Jr [a]

Aniruddh D. Patel [b]

[a] Department of Speech, Language and Hearing Sciences, Boston University, Boston, MA;
[b] Department of Psychology, Tufts University, Malden, MA

Key words: Musicians, speech in noise, informational masking, individual differences, spatial hearing

[1] To whom correspondence should be addressed. E-mail: jswamy@bu.edu.

Classification – Neuroscience

Date originally submitted: November 10, 2014

Date of resubmission: May 12, 2015

SI Results

A key feature of our design was the manipulation of degree of informational masking (IM) between speech and background noise, while keeping the amount of energetic masking (EM) relatively constant. This was achieved by measuring the ability to identify a speech target in the presence of two symmetrically placed speech maskers that were either highly intelligible and confusable with the target (forward maskers: high in IM) or time-reversed and unintelligible and thus less confusable with the target (reversed maskers: lower in IM). In the past, the evidence for the equivalent EM produced by forward- and time-reversed speech maskers has taken the form of indirect second-language comparisons [1] or by substituting envelope-modulated noise for the masker talkers [2]. In both of those studies in contrast to early work by [3] the EM produced by reversed speech was equal to or greater than that produced by natural speech. However, these studies were rather indirect and did not provide any direct evidence at the most relevant physiological level of processing; i.e., the cochlea and auditory nerve [4].

Here, we used a phenomenological auditory-nerve (AN) model [5] to verify that, for the stimuli used in this study, forward and reversed maskers produced similar EM of the target temporal features. The temporal coding of target features was analyzed in terms of strength of coding of target temporal fine structure (TFS, the rapid variations with rate close to the characteristic frequency of the AN fiber) and envelope (ENV, the relatively slow variations in amplitude over time) in the presence of forward or reversed maskers. We hypothesized that, if the forward and reversed maskers produced similar EM, the strength of coding of target temporal features (TFS and ENV) in the auditory-nerve will be similar for target speech degraded by forward or reverse speech maskers.

The target speech was added to two different exemplars of forward or reversed maskers at ten different signal-to-noise ratios (SNR=20, 10, 5, 0, -2, -5, -7, -10, -15 and -20 dB). Stimuli were resampled to 100 kHz prior to presentation to the AN model to obtain the spike times. Eight high-spontaneous-rate AN fibers with characteristic frequencies (CFs) varying from 200 Hz to 8 kHz were selected. The CFs of the fibers were 250, 500, 750, 1000, 1500, 2000, 4000 and 8000 Hz.

For each AN fiber, the salience of target-speech related TFS and ENV coding following degradation (i.e., due to forward or reversed maskers) was quantified by computing neural cross-correlation (ρ_{TFS} and ρ_{ENV}) between target speech in quiet (baseline) and target+maskers at different signal-to-noise ratios (SNRs). The methodological details for computing these neural cross-correlational coefficients are provided in refs [6,7] and briefly described below.

Fig. S1 demonstrates the use of neural cross-correlational coefficients (ρ_{TFS} and ρ_{ENV}) to show the effect of masking noise on the neural coding of target temporal features (ENV and TFS). Neural cross correlation coefficients for ENV and TFS were computed between target speech in quiet (column 1 in Fig. S1) and target + forward maskers at 0 dB SNR (column 2 in Fig. S1). Separate metrics for TFS and ENV were computed using shuffled auto- and cross-correlograms [8-10]. Shuffled auto correlograms are computed by tallying spike intervals across stimulus repetitions (rather than within repetitions), and yield more robust characterizations of temporal responses than classic all-order interval histograms [11]. Normalized shuffled auto correlograms are plotted as function of delay (or inter-spike interval) and are much like auto-correlation functions (Fig. S1A-B, dark lines).

TFS and ENV coding can be separated by comparing the responses to a stimulus and its polarity-inverted pair (e.g., A+ with A-) [8-10]. Polarity inversion acts to invert the TFS, but does

not affect ENV. Cross-polarity correlograms are computed by comparing spikes from A+ and A- (Fig. S1A-B, thin lines). To emphasize TFS coding, difcors were computed as the difference between the shuffled auto correlogram (original ENV, original TFS; thick line in Fig. S1A) and the cross-polarity correlogram (original ENV and inverted TFS; thin line in Fig. S1A), where the difcor peak height quantifies the strength of TFS coding. To quantify ENV coding, sumcors were computed as the average of the shuffled auto correlogram and the cross-polarity correlogram. The third column of Fig. S1 illustrates the use of shuffled cross correlograms (SCC) to quantify the similarity between spike trains in response to different stimuli (e.g., intact speech in quiet (A) and degraded speech (B)). Cross-stimulus correlograms [e.g., SCC(A+, B+), thick line in Fig. S1C] and cross-stimulus, cross-polarity correlograms [e.g., SCC(A+,B-), Fig. S1C, thin line] were computed to facilitate the separation of TFS and ENV cross correlations by using difcors and sumcors, respectively.

Neural cross-correlation coefficients [6] ranging between 0 and 1 were computed by comparing the degree of response similarity (column 3 of Fig. S1) to the degree of temporal coding for each stimulus individually (columns 1 and 2 of Fig. S1). The cross-correlation coefficient for TFS was computed from the difcor peak heights as

$$\rho_{TFS} = \frac{difcor_{AB}}{\sqrt{difcor_A \times difcor_B}}.$$

Likewise, the neural cross-correlation coefficient for ENV was computed from the sumcor peak heights (after subtracting the baseline value of 1) as

$$\rho_{ENV} = \frac{(sumcor_{AB} - 1)}{\sqrt{(sumcor_A - 1) \times (sumcor_B - 1)}}.$$

For the single-fiber responses in Fig. S1, the temporal coding of TFS and ENV for target speech in quiet and for target speech degraded by forward speech maskers at 0 dB SNR was quite similar (ρ_{TFS} =0.60 and ρ_{ENV} =0.68).

The effects of forward and reversed maskers on the neural coding of target ENV and TFS are shown in Fig. S2. A total of 3200 sets of neural cross-correlation coefficients were computed (8 AN fibers x 10 SNRs x 2 masker types x 20 repetitions). TFS coding of fibers with CF > 2000 Hz were not included due to roll-off in phase locking. To simplify the data analysis and for clarity of presentation, for each SNR, cross-correlation coefficients were averaged across all AN fibers. Across CFs, the overall trends in neural coding were similar, as shown by small standard-error bars (Fig. S2). Mean TFS and ENV coding across CFs was identical for forward and reversed maskers for all SNRs suggesting that both these maskers produced identical EM patterns of target temporal features.

SI References

1 Rhebergen, K. S., Versfeld, N. J. & Dreschler, W. A. Release from informational masking by time reversal of native and non-native interfering speech. *J Acoust Soc Am* **118**, 1274-1277 (2005).

2 Marrone, N., Mason, C. R. & Kidd, G. The effects of hearing loss and age on the benefit of spatial separation between multiple talkers in reverberant rooms. *J Acoust Soc Am* **124**, 3064-3075 (2008).

3 Dirks, D. D. & Bower, D. R. Masking effects of speech competing messages. *Journal of Speech, Language and Hearing Research* **12**, 229-245 (1969).

4 Durlach, N. I. *et al.* Note on informational masking. *J Acoust Soc Am* **113**, 2984-2987 (2003).

5 Zilany, M. S. A. & Bruce, I. C. Representation of the vowel /ε/ in normal and impaired auditory nerve fibers: model predictions of responses in cats. *J Acoust Soc Am* **122**, 402-417 (2007).

6 Heinz, M. G. & Swaminathan, J. Quantifying envelope and fine-structure coding in auditory nerve responses to chimaeric speech. *J Assoc Res Otolaryngol* **10**, 407-423 (2009).

7 Swaminathan, J. & Heinz, M. G. Psychophysiological analyses demonstrate the importance of neural envelope coding for speech perception in noise. *J Neurosci* **32**, 1747-1756 (2012).

8 Joris, P. X. Interaural time sensitivity dominated by cochlea-induced envelope patterns. *J Neurosci* **23**, 6345-6350 (2003).

9 Joris, P. X., Louage, D. H., Cardoen, L. & van der Heijden, M. Correlation index: a new metric to quantify temporal coding. *Hear Res* **216-217**, 19-30 (2006).

10 Louage, D. H., Van Der Heijden, M. & Joris, P. X. Temporal properties of responses to broadband noise in the auditory nerve. *J Neurophysiol* **91**, 2051-2065 (2004).

11 Ruggero, M. A. Response to noise of auditory nerve fibers in the squirrel monkey. *J Neurophysiol* **36**, 569-587 (1973).

SI Figure Legends

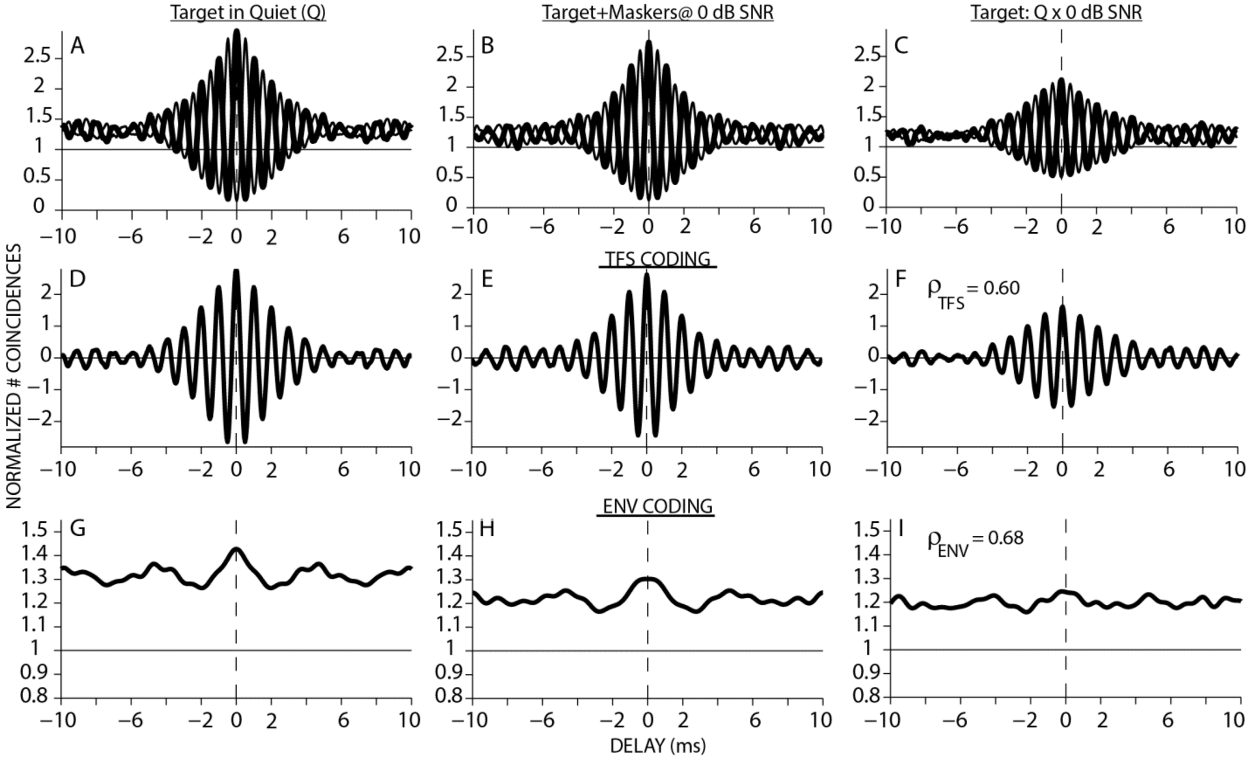

Fig. S1. [1 *column*] Correlogram analyses to quantify the neural coding of ENV and TFS in noise-degraded speech. Columns 1 and 2 show temporal coding of target speech in quiet (Q) and for target + forward maskers at 0 dB SNR, respectively; column 3 illustrates the similarity in temporal coding between these two conditions. Panels A and B: Normalized shuffled auto correlograms (thick line) and cross-polarity correlograms (thin line). Panel C: Shuffled cross-stimulus correlogram (thick line) and cross-polarity, cross-stimulus correlogram (thin line). Panels D–F represent within and across-stimulus TFS coding, with ρ_{TFS} shown in F. Panels G–I represent within and across-stimulus ENV coding, with ρ_{ENV} shown in I. Fiber CF=1000 Hz.

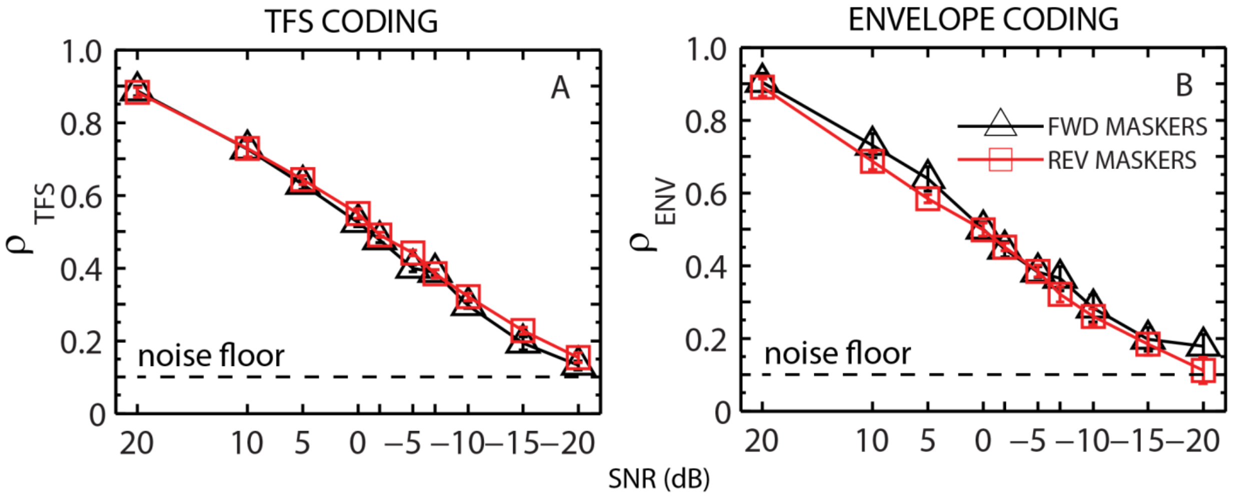

Fig. S2. ***[1.5 columns]*** Neural coding of target envelope and temporal fine structure was similar for forward and reversed maskers at all SNRs. Hence, forward and reversed maskers produce identical temporal/energetic masking of target features at all SNRs. Panels A and B show mean neural coding of TFS and ENV for target degraded by forward (FWD) and reversed (REV) maskers.
